# Unveiling the role of knowledge within the concept of physical literacy and its empirical links to physical activity in children

**DOI:** 10.1016/j.jesf.2026.200524

**Published:** 2026-09-23

**Authors:** Tizian Schuck, Johannes Carl, Susanne Tittlbach

**Affiliations:** a Bayreuth Center of Sport Science, Chair of Social and Health Sciences, University of Bayreuth, Universitätsstraße 30, Bayreuth, 95447, Germany; b Department of Sport Science and Sport, Chair of Education in Sport, Friedrich-Alexander-University of Erlangen-Nürnberg, Gebbertstraße 123b, Erlangen, 91058, Germany; c Department of Sport and Human Movement Sciences , University of Vienna, Auf der Schmelz 6a, Wien, 1150, Austria

**Keywords:** Cognitive dimension, Daily behavior, Health-related knowledge, Mediation analysis

## Abstract

**Background:**

Physical literacy is a key concept for promoting lifelong physical activity, integrating an individual's motivation, confidence, physical competence, knowledge, and understanding. While research has predominantly focused on the physical and affective dimensions of physical literacy, the cognitive dimension remains empirically underexplored. This study analyses (a) how the cognitive dimension and physical activity are associated in German primary school children and (b) whether the other dimensions of physical literacy mediate this association.

**Methods:**

Based on a cross-sectional survey of 549 s-grade students (mean age = 8.4, SD = .5), physical literacy was assessed using motor tests (GMT 6-18) and validated questionnaire items on physical activity (number of active days, number of sports and exercises performed), physical self-concept, motivation, and enjoyment. The cognitive dimension was operationalized through two open-ended questions on declarative and procedural knowledge, coded independently by two raters (*κ* > .90). Structural equation modeling with bootstrapped mediation analysis was used to examine the direct and indirect associations between the cognitive dimension and children's physical activity.

**Results:**

Declarative knowledge showed a small but significant direct association with the number of active days (*β* = .10), with a complementary mediation via motor abilities (*β* = .12). Procedural knowledge followed an ‘indirect-only pattern’ on number of sports and exercises performed via the affective dimension (*β* = .09).

**Conclusion:**

Knowledge showed limited direct associations with physical activity but indirect pathways via physical and affective dimensions, highlighting the potential of embedding cognitive content in active learning experiences. Promoting physical literacy in physical activity settings should address all its dimensions and embed knowledge acquisition within physical activity.

## 1 Introduction

Regular physical activity is widely recognized for its numerous health benefits and is, therefore, essential for promoting health and well-being across the lifespan.^1^ Nevertheless, physical inactivity remains a major public health challenge, including among children.^2^ Since childhood represents a critical period for establishing long-term physical activity habits,^3^^,^^4^ understanding how these behaviors can be fostered from an early age is critical from a health promotion perspective.

In recent years, physical literacy has emerged as a key concept for promoting lifelong engagement in physical activity.^5^ It is commonly defined as “the motivation, confidence, physical competence, knowledge, and understanding to value and take responsibility for engaging in physical activities for life”.^6^ While Whitehead's^7^ original conceptualization of physical literacy is grounded in a monistic understanding of the individual, subsequent international frameworks have adapted the concept to their respective cultural and educational contexts.^8^ The majority of the various existing concepts of physical literacy consist of physical, cognitive and psychological-affective dimensions,^9^ sometimes complemented by additional aspects, such as physical activity participation (Canadian model^10^), social skills (Australian model^11^), or sensory-perceptual attributes (Chinese model^12^). In Germany, no universally accepted model or definition exists.^13^ Therefore, the present study adopts Whitehead's^7^ conceptualization as its theoretical foundation and draws on the Canadian model^10^ for empirical operationalization. Within the diverse spectrum of different understandings of physical literacy,^14^ this study explicitly follows other researchers with a pragmatist stance who attempt to empirically capture aspects of physical literacy without fully detaching from its key tenets.^9^ Following the Canadian model,^10^ the daily physical activity behavior is conceptualized as a behavioral dimension that is dynamically interrelated with the cognitive, physical, and affective dimensions rather than merely representing an outcome of physical literacy.^15^

Although numerous instruments have been developed to assess physical literacy for specific age groups, the methodological quality of these measurements varies considerably.^16^^,^^17^ While most instruments demonstrate promising psychometric properties for capturing the physical and affective dimensions, the cognitive dimension often reveals weaker assessment quality.^9^^,^^18^^,^^19^ The majority of assessments often attempt to capture the cognitive dimension across the entire age span, which leads to high item complexity and can easily overburden younger children.^10^^,^^11^

In Germany, assessment tools for physical literacy are lacking and empirical studies remain scarce.^13^ Initial efforts include the development of a new questionnaire^20^ and the linguistic adaptation of international assessments.^21^ First empirical findings indicate that the cognitive dimension appears less influential in childhood^22^ and its role in predicting physical activity behavior may be smaller than that of other physical literacy dimensions.^23^

Despite these findings, knowledge as part of the cognitive dimension is widely regarded as a central but underexplored component of physical literacy.^24^ In sport pedagogical and didactical perspectives, knowledge is considered a fundamental prerequisite for developing competence and enabling individuals to act appropriately in diverse movement situations. Accordingly, the aim of physical education is not limited to the acquisition of motor skills but includes the development of an *ensemble of knowledge and abilities* that enables students to cope with complex demands in sport and movement context.^25^^,^^26^ Within this understanding, knowledge represents an essential foundation of *Handlungsfähigkeit* [capacity to act], a central concept in German physical education that integrates knowing, doing, and reflecting as inseparable dimensions of educational processes.^27^

Within this perspective, knowledge relevant for physical activity is not restricted to factual understanding but also includes the ability to apply knowledge appropriately in specific situations. For example, the framework of sport-related health competence^28^ distinguish between declarative knowledge (e.g., knowledge about the health-related effects of physical activity; *effect knowledge*) and procedural knowledge (e.g., knowledge about how to design and regulate actual physical activity; *action knowledge*). They emphasize that competence does not result from knowledge alone but from the targeted and situation-appropriate application of knowledge. Similarly, sport pedagogical competence models highlight the interplay between knowledge and action as a basis for competent participation in sport and physical activity^29^, ^30^, ^31^.

Accordingly, the present study distinguishes between declarative and procedural knowledge as complementary components of the cognitive dimension of physical literacy. Both forms of knowledge may contribute to informed decision-making, reflective engagement, and sustained participation in physical activity. Recent conceptual models of physical literacy further emphasize its dynamic and interrelated nature.^32^ More specifically, the model positions knowledge as a supporting component that informs the development of physical and affective aspects of physical literacy. In turn, these interconnected aspects provide plausible pathways through which knowledge may become associated with physical activity.^32^ This perspective corresponds closely with Whitehead's^7^ conceptualization, highlighting the role of knowledge and understanding in linking movement, health, and lifelong engagement in physical activity. Moreover, it resonates with modern conceptualizations of ‘literacy’, which extend beyond basic comprehension to include the critical and reflective processing and application of knowledge.^33^^,^^34^

Knowledge as one aspect of the broader cognitive dimension, is considered both a prerequisite for physical performance and a driver of transfer, innovation, and critical reflection.^24^^,^^35^ Building on these considerations, the present study investigates the role of knowledge within physical literacy in German primary school children. Specifically, we investigated the following research questions:

(1) Is knowledge directly associated with physical activity behavior in children?
(2) Do physical and affective dimensions mediate this potential association?

## 2 Methods

### 2.1 Participants and study design

This cross-sectional study was part of the project *Bewegungswelt Oberfranken – aktive und bewegungskompetente Kinder* [*Movement World Upper Franconia – Active and physically literate children*]. Participants were 549 s-grade children (mean age = 8.4 ± .5 years; 49 % girls) from 23 participating primary schools located in both urban and rural areas of the city of Bayreuth (Bavaria, Germany). All students who were present at the participating schools on the assessment days and fulfilled the inclusion criteria (enrollment in second grade; absence of medical conditions or physical impairments) were invited to participate.

Data collection took place in June 2024 during regular school hours under standardized conditions. Motor tests were administered in school gymnasiums in small groups. The assessment procedures followed the standardized instructions and administration guidelines of the GMT 6–18^36^. Prior to testing, all children participated in a standardized warm-up session to prepare them physically for the assessments. To reduce potential order effects and fatigue-related influences, the sequence of motor tasks varied across groups. All assessments were conducted by trained personnel who received standardized instructions regarding test administration, scoring procedures, and the provision of task demonstrations. Questionnaires were completed individually in the classroom with support from trained staff and teachers. Parental follow-up reports on children's physical activity were collected immediately after the school-based assessments within the same school year (July – September 2024) to obtain a more reliable estimate on children's self-reports on physical activity. The extended period reflected the organization of parental questionnaire distribution and return procedures. Written informed consent was obtained from legal guardians prior to participation.

### 2.2 Theoretical model

The study is guided by a theoretically informed model of physical literacy,^10^ conceptualizing physical literacy as a multidimensional construct comprising cognitive, physical, affective, and behavioral dimensions.

The present analysis focuses on associations between the cognitive and behavioral dimensions. The cognitive dimension was operationalized through declarative and procedural knowledge, and the behavioral dimension via indicators of physical activity participation (active days; number of sports and exercises performed). Based on conceptual models suggesting that knowledge and understanding are linked to the development of physical competence and affective attributes relevant for physical activity engagement,^32^ the empirical model examines potential indirect pathways via motor abilities and a latent affective factor (physical self-concept, motivation, and enjoyment).

Fig. 1 illustrates the hypothesized model; circles indicate theoretical assumptions and squares empirical operationalizations.

**Fig. 1:**
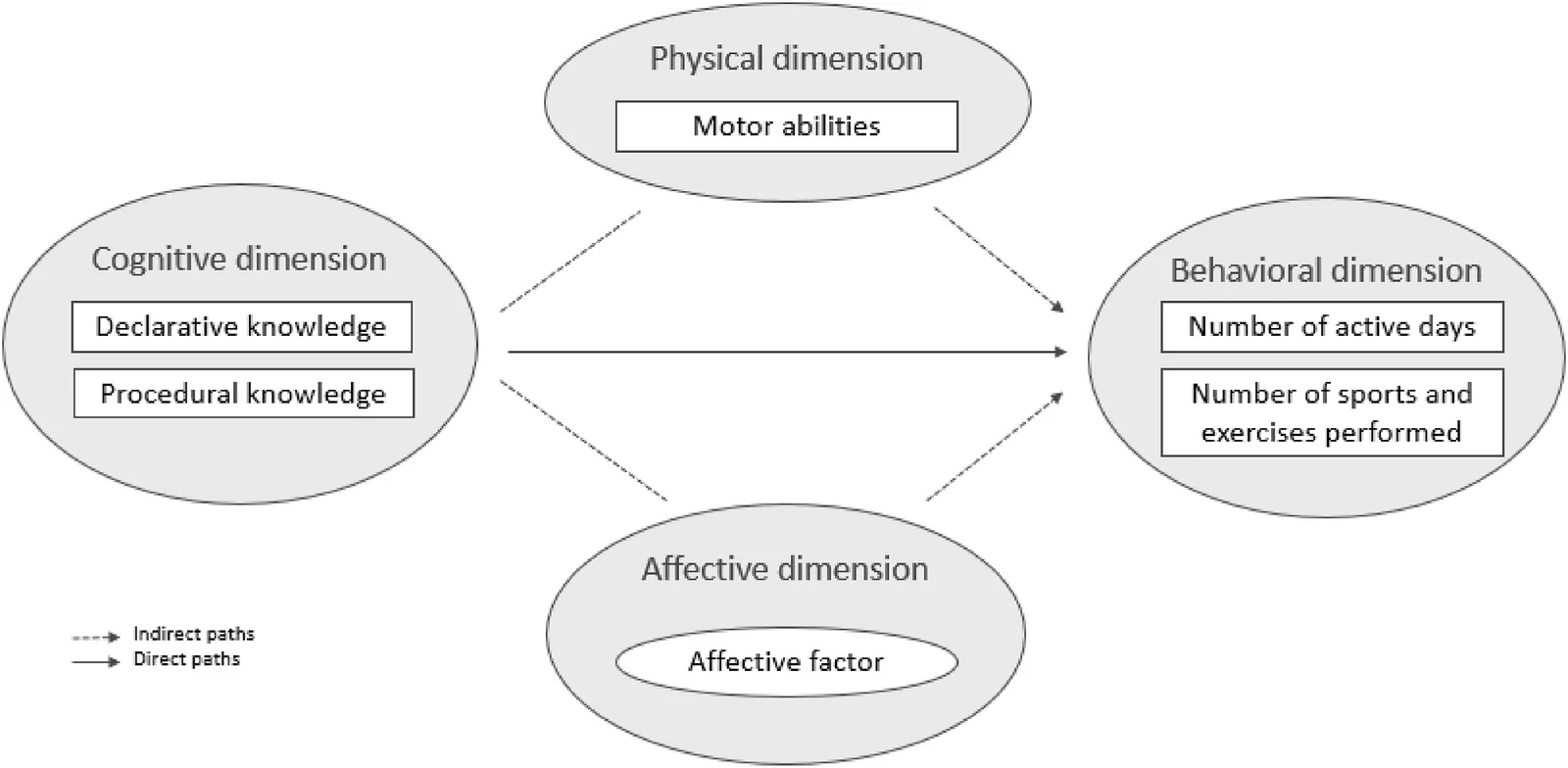
Simplified representation of the hypothesized relationships within physical literacy.

### 2.3 Variables and measurements

To operationalize the cognitive dimension of physical literacy, the present study focused specifically on health-related knowledge as an initial operationalization, including declarative (effect) and procedural (action) knowledge^28^,^31^*.* It should be noted that the cognitive dimension may encompass additional aspects beyond knowledge, which were not included in the current analysis.

Declarative knowledge was assessed using an open-ended item based on Krenz et al.^20^ In line with their approach, an open-ended format was selected to ensure age-appropriate access to children's spontaneous knowledge representations without restricting responses through predefined options. Children were asked: *“What signs in your body tell you that you have been physically active or have been exercising?“.* Responses were coded into four categories: physical reaction (e.g., sweating, feeling hot), physical adaptation (e.g., sore muscles), perceived effort (e.g., feeling tired), and well-being (e.g., feeling relaxed).

Procedural knowledge was assessed with an open-ended item adapted from Longmuir et al.^37^ Children were asked: *“If you wanted to improve in sport, what would you do?“.* Responses were coded into four categories: social support (e.g., asking friends, parents, or coaches), amount of time (e.g., training every day), effort (e.g., practicing deliberately), and training principles (e.g., structured progression from beginner to advanced levels).

For both knowledge items, categories were developed inductively through an iterative consensus process within the research team, while being theoretically informed by previous work.^20^^,^^31^^,^^37^ Coding was performed independently by two raters (κ > .90). Detailed coding schemes are provided in the supplementary material (Table S1–S4).

Standardized and age-appropriate operationalizations were selected for the physical, affective, and behavioral dimensions. Details regarding item numbers, response formats, scoring procedures, and reliability estimates are provided in Table 1. To operationalize the behavioral dimension, activity questions for number of active days and number of sports and exercises performed were used.^38^ The affective dimension was operationalized through the physical self-concept,^39^ motivation subscales,^40^ and enjoyment subscale.^41^ The physical dimension was operationalized using a standardized motor test battery (GMT 6-18^36^). The GMT 6–18 is a standardized motor competence assessment for children and adolescents, comprising tasks assessing different domains of motor performance, including coordination, speed, flexibility, and strength/endurance. In the present study, seven subtests were included. Following the test manual, raw scores were standardized using z-scores and aggregated into a composite motor ability score. The push-up test was not included because muscular strength was already represented by the sit-up test within the battery. This decision was made to reduce redundancy and minimize testing burden for second-grade children.

**Table 1:** Description of the operationalization of physical literacy.

Dimension of physical literacy	Manifest variable	Instrument	Score range	Validity	Reliability/Internal consistency (α)
Physical	Motor abilities	GMT 6–18^36^, 7 motor tests), z-transformed, composite score	Standardized z-scores	Criterion validity supported by teacher ratings (r = .69)	Test–retest r = .52 – .94
Behavioral	Number of active days	MoMo activity question^38^	0 – 7	Criterion validity supported by accelerometer (r = .29)	Weighted kappa coefficient and ICC for total index: .66 – .68
	Number of sports and exercises performed	MoMo activity question^38^	0 – n	Criterion validity supported by accelerometer (r = .29)	Weighted kappa coefficient and ICC for total index: .66 – .68
Affective	Physical self-concept	Questionnaire without subscale attractiveness,^39^ 6 subscales with 3 items each	1 – 4	Criterion validity supported by associations with gender, motor abilities, and BMI	α = .57 – .82
	Motivation	Subscales intrinsic & identified motivation,^40^ 3 items each	1 – 4	Construct validity confirmed (r = .37**)	α = .66 – .83
	Enjoyment	Subscale enjoyment,^41^ 3 items	1 – 4	High construct reliability (λ = .50 – .88; ω = .91)	Test–retest r = .81, α = .86
Cognitive	Declarative knowledge	Question oriented on Krenz et al.^20^	Open-ended	/	/
	Procedural knowledge	Question oriented on Longmuir et al.^37^	Open-ended	/	/

**α** Cronbach's Alpha **ICC** Interclass-coefficient, **GMT** German Motor test.

### 2.4 Statistical analysis

All analyses were carried out in *R (*v 4.4.3, R Foundation for Statistical Computing, Vienna, Austria). The complete R code used for data preparation, preliminary analyses, multidimensional scaling (MDS), confirmatory factor analysis (CFA), and structural equation modeling (SEM) is publicly available via the Open Science Framework (https://osf.io/4hqp5/files/osfstorage/6ab4f4610dc60920bcd32aad).

Prior to the main analyses, data were screened for completeness, plausibility, and potential outliers. Descriptive statistics were calculated for all study variables, and distributional characteristics were examined using skewness and kurtosis values as well as visual inspection of variable distributions. Potential outliers and influential observations were assessed using regression-based diagnostic procedures, including residual-leverage plots.

Multidimensional scaling (MDS) was used to explore the conceptual structure of physical literacy items (see Fig. S1 in the supplementary material). Confirmatory factor analyses (CFA) assessed the model fit using Comparative Fit Index (*CFI*) > .95, Root Mean Square Error of Approximation (*RMSEA*) ≤ .06 with 90 % confidence intervals (*CI*), and Standardized Root Mean Square Residual (*SRMR*) ≤ .08, with Bollen–Stine bootstrap for the evaluation of the overall fit.^42^

Structural equation modeling with the *lavaan* package^43^^,^^44^ tested (1) direct associations between knowledge and behavioral indicators, (2) indirect associations mediated via motor abilities and the latent affective factor. To comprehensively examine the relationships for (2), total, total indirect, and partial indirect associations were computed. Total associations reflect the overall relationship (direct + indirect), total indirect associations quantify the contribution of the mediators, and partial indirect associations separate the associations of each mediator. Factor loadings (*λ*) above .50 were considered acceptable, with values above .70 indicating good indicator reliability.^45^^,^^46^ Parameter estimates were reported as standardized regression coefficients (*β*), interpreted as small (≈.10), medium (≈.30), or large (≥.50) effects.^47^ Missing data were handled using full information maximum likelihood, and indirect associations were estimated with 5000 non-parametric bootstrap resamples and bias-corrected and accelerated 95 % confidence intervals^48^, ^49^, ^50^.

## 3 Results

### 3.1 Descriptive analyses

Descriptive statistics for all dimensions of physical literacy are presented in Table 2. Mean scores were generally slightly above the midpoint of the response scales. Prior to the main analysis, data screening procedures indicated satisfactory data quality. No deviations from distributional assumptions were observed, and no problematic outliers remained after the screening procedure.

**Table 2:** Descriptive statistics of each physical literacy dimension for the combined sample and by sex.

	Boys (n = 281)	Girls (n = 268)	Total (n = 549)
**Knowledge dimension**			
Declarative Knowledge	M = 1.97, SD = .68, R = 1.00 – 3.00	M = 2.00, SD = .71, R = 1.00 – 3.00	M = 1.98, SD = .69, R = 1.00 – 3.00
Procedural Knowledge	M = 2.26, SD = .69, R = 1.00 – 3.00	M = 2.30, SD = .79, R = 1.00 – 3.00	M = 2.28, SD = .74, R = 1.00–3.00
**Physical dimension**			
Motor abilities	M = 101.35, SD = 5.73, R = 79.83 – 112.92	M = 100.45, SD = 5.66, R = 80.97 – 117.44	M = 100.91, SD = 5.71, R = 79.83 – 117.44
**Affective dimension**			
Physical self-concept	M = 3.51, SD = .41, R = 1.78 – 4.00	M = 3.31, SD = .44, R = 1.72 – 4.00	M = 3.42, SD = .43, R = 1.72 – 4.00
Motivation	M = 3.47, SD = .49, R = 1.00 – 4.00	M = 3.30, SD = .54, R = 1.50 – 4.00	M = 3.39, SD = .52, R = 1.00–4.00
Enjoyment	M = 3.78, SD = .38, R = 2.00 – 4.00	M = 3.69, SD = .44, R = 2.00 – 4.00	M = 3.74 SD = .41, R = 2.00 – 4.00
**Behavioral dimension**			
Number of active days	M = 4.47, SD = 1.75, R = .00 – 7.00	M = 4.34, SD = 1.74, R = .00 – 7.00	M = 4.41, SD = 1.75, R = .00 – 7.00
Number of sports and exercises performed	M = 2.41, SD = 1.23, R = 1.00 – n	M = 2.28, SD = 1.36, R = .00 – 8.00	M = 2.35, SD = 1.29, R = .00 – 8.00

**M** Mean, **SD** Standard deviation, **R** Range.

### 3.2 Conceptual and empirical item placement

MDS revealed four conceptually distinct but interrelated clusters, aligned with the cognitive, physical, affective, and behavioral dimensions of physical literacy. Visually, the closest proximity was observed between the cognitive and behavioral dimensions, in contrast to the greater distance between the physical and affective dimensions (see Fig. S1 in the supplementary material).

CFA indicated an acceptable model fit across most indices (*χ*^*2*^*/df* = 2.84, Bollen–Stine bootstrap *p* = .02, *CFI* = .97, *RMSEA* = .06, 90 % *CI* [.03, .08], *SRMR* = .03). Standardized factor loadings for the affective factors were acceptable (*λ* = .68 - .71). Significant covariances were observed between the behavioral indicators (*cov* = .22, *p* < .001) and between the physical and affective dimensions (*cov* = .25, *p* < .001). Significant associations are illustrated in Fig. 2.

**Fig. 2:**
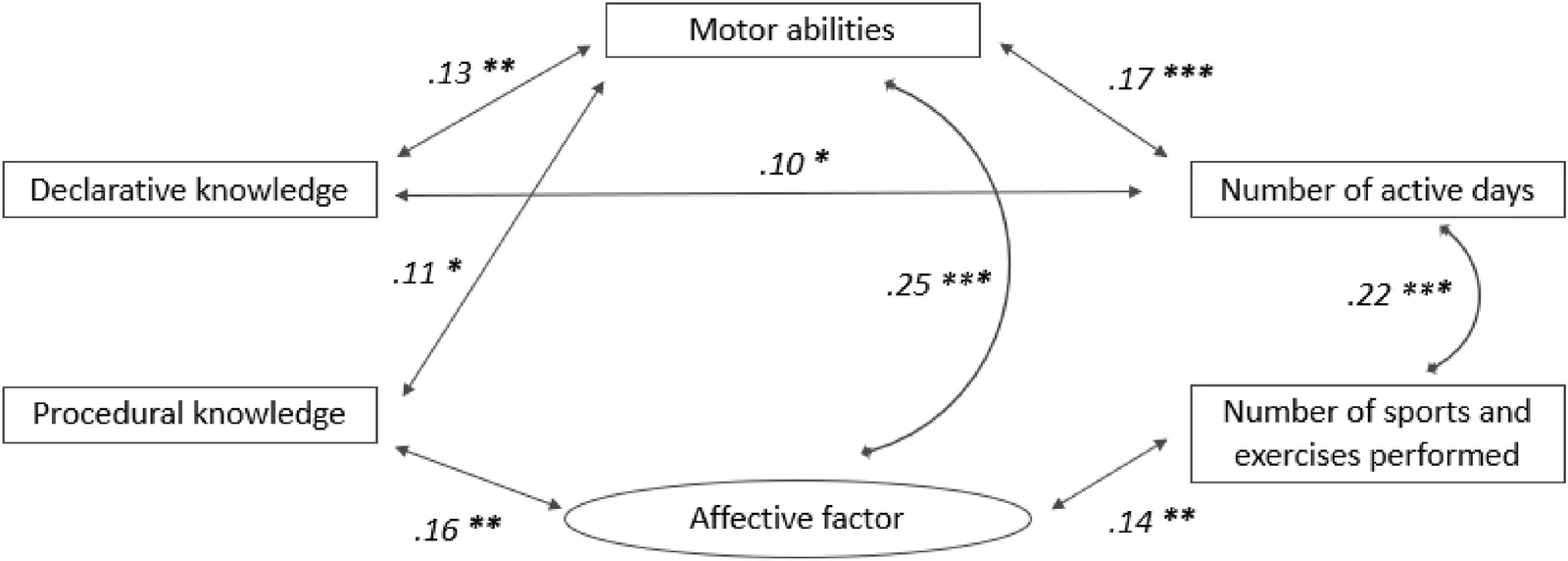
Estimated empirical model with significant associations (*p < .05, **p < .01, ***p < .001).

### 3.3 Associations between knowledge and physical activity behavior

All associations between the operationalization's of physical literacy are shown in Table 3. Declarative knowledge was positively associated with the number of active days per week *(β* = .10, *p* < .05). Total associations (*β* = .12, *p* < .01) indicated a stronger direct link than the partially indirect link mediated by motor abilities (*β* = .02, *p* < .05). No mediation was found via the affective dimension. In contrast, declarative knowledge was not significantly associated with the number of sports and exercises performed (*β* = −.04, *p = .38*).

**Table 3:** Associations between knowledge and behavioral indicators.

Predictor	Outcome	Path type/Mediator	Statistics *b (SE), 95 % CI, β (std. all), p*
Declarative Knowledge	Number of active days	Direct	.40 (.17), [.09, .74], .10 *
		Total (direct + indirect)	.48 (.17), [.16, .83], .12 **
		Total indirect (via both mediators)	.08 (.04), [.00, .18], .02 ^ns^
		Partial indirect (via motor abilities)	.09 (.04), [.03, .18], .02 *
		Partial indirect (via affective dimension)	.00 (.02), [-.06, .02], .00 ^ns^
	Number of sports and exercises performed	Direct	−.12 (.13), [-.37, .13], −.04 ^ns^
		Total (direct + indirect)	−.11 (.13), [-.04, .14], −.04 ^ns^
		Total indirect (via both mediators)	.01 (.03), [-.05, .07], .00 ^ns^
		Partial indirect (via motor abilities)	.02 (.02), [.00, .08], .01 ^ns^
		Partial indirect (via affective dimension)	−.01 (.02), [-.06, .03], .00 ^ns^
Procedural Knowledge	Number of active days	Direct	.23 (.21), [-.18, .63], .05 ^ns^
		Total (direct + indirect)	.37 (.20), [-.03, .76], .08 ^ns^
		Total indirect (via both mediators)	.13 (.06), [.03, .28], .03 *
		Partial indirect (via motor abilities)	.08 (.05), [.02, .12], .02 ^ns^
		Partial indirect (via affective dimension)	.02 (.02), [.00, .08], .01 ^ns^
	Number of sports and exercises performed	Direct	.24 (.15), [-.05, .53], .07 ^ns^
		Total (direct + indirect)	.34 (.15), [.05, .64], .10 *
		Total indirect (via both mediators)	.10 (.05), [.03, .21], .03 *
		Partial indirect (via motor abilities)	.05 (.04), [-.02, .16], .01 ^ns^
		Partial indirect (via affective dimension)	.08 (.04), [.02, .18], .02 *

***b*** Unstandardized estimate, ***SE*** Standard error, ***CI*** 95 % Confidence interval, ***β*** Standardized coefficient, **ns** not significant, *p < .05, **p < .01, ***p < .001.

Procedural knowledge showed no direct association with the number of active days, but total indirect associations via both mediators (*β* = .03, *p* < .05). For the number of sports and exercises performed, procedural knowledge was associated both in total (*β* = .10, *p* < .05) and through partial mediation via the affective dimension (*β* = .02, *p* < .05), whereas motor abilities showed a smaller, non-significant contribution (*β* = .01, *p* = .24).

## 4 Discussion

The present study examined whether knowledge is associated with physical activity behavior in children, whether these associations are mediated by physical and affective dimensions. Declarative knowledge showed a complementary mediation pattern, with a significant direct association and a small additional indirect association via motor abilities. Procedural knowledge followed an indirect-only pattern, being linked to activity indicators exclusively through indirect pathways (i.e., via the affective dimension).

Declarative and procedural knowledge showed distinct associations with physical activity behavior, supporting the relevance of differentiating between these two forms of knowledge within the cognitive dimension of physical literacy. Declarative knowledge – operationalized via children's understanding of their body signals of physical activity – was associated with overall active days and may be acquired early through everyday socialization.^31^^,^^51^ In contrast, procedural knowledge – understanding how to perform or improve activity – is cognitively more demanding and may only influence behavior when embedded in motor skill development and self-regulation.^31^^,^^52^ This distinction helps to explain previous inconsistent findings, where knowledge was often treated as a unidimensional construct.^22^^,^^23^ Overall effect sizes were small, reinforcing that knowledge alone is insufficient to decisively drive activity. Prior studies similarly indicated that general awareness of physical activity benefits is commonly known, yet only more specific or detailed knowledge shows meaningful associations with behavior^53^, ^54^, ^55^.

Indirect pathways provided additional insight into how knowledge operates within physical literacy. Previous research has emphasized that the relationship between physical literacy and physical activity in children is dynamic and reciprocal rather than unidirectional.^56^ Studies have consistently reported positive associations between overall physical literacy and physical activity among primary school children.^57^^,^^58^ However, previous studies have commonly examined physical literacy as a multidimensional but relatively aggregated construct. The present study extends this perspective by empirically examining declarative and procedural knowledge as complementary expressions of knowledge within physical literacy. Declarative knowledge was linked to activity directly and indirectly via motor abilities, although the direct pathway was stronger. It is possible that the associations follow a different sequence, such as knowledge is first linked with activity, which in turn affects motor abilities. Children who understand the health benefits of activity may thus be more likely to engage in physical activity, which could further support motor abilities. These findings extend prior research that conceptualized physical literacy as a global construct and identified indirect pathways through competence, fitness, and motivation.^59^^,^^60^ In contrast, procedural knowledge was primarily associated with behavioral indicators through indirect pathways, most consistently via the affective dimension and the number of sports and exercises performed. Consistent with embodied learning perspectives,^35^ this suggests that associations between the procedural knowledge and behavior are stronger when embedded in bodily and affective experiences. This aligns with the Information-Motivation-Behavioral Skills model,^61^^,^^62^ which posits that information alone is insufficient to elicit behavior change unless accompanied by motivational and behavioral skills. In this sense, behavioral skills may represent the enacted expression of procedural knowledge – that is, the capacity to translate embodied understanding into physical activity behavior. In line with this, physical education that does not actively involve students or miss to connect the delivery of health-related knowledge to physical experiences lead to a lower development of health-related knowledge.^30^ The present findings underscore why separating the delivery of knowledge from physical experiences may hinder children's engagement in physical activity. By empirically distinguishing the differential pathways, this study refines current models of physical literacy by suggesting that knowledge types are associated with distinct yet complementary patterns of relations. Future research should replicate these findings in experimental or longitudinal designs to further validate these mechanisms and examine their generalizability across settings.

Although the physical and affective dimensions exhibited distinct pathways in relation to behavioral indicators, this statistical separability does not imply that they operate independently. Rather, the indirect pathways indicate that the dimensions are interdependent, consistent with a monistic understanding of physical literacy.^7^ In this regard, the present study did not examine *whether* bur rather *how* the different aspects of physical literacy interact to form a monistic entity.

Differences in pathways may reflect developmental factors: in childhood, some dimensions may be less developed than others. Biographical mapping studies suggest that the relative importance and developmental trajectories of individual dimensions vary across the life course, supporting the notion that the influence of different dimensions on physical activity is developmentally dynamic.^22^ Future longitudinal studies are needed to further understand how cognitive, physical, and affective dimensions co-develop and link with behavior over time.

These findings support the practical perspective that knowledge is likely most relevant when considered alongside physical and affective dimensions. Declarative knowledge can be reinforced through playful activity routines connecting health content to everyday activity (e.g., understanding why and under which conditions exercise can be health-enhancing), while procedural knowledge requires opportunities for children to apply, test, and reflect on their own strategies in concrete movement situations, such as games, obstacle courses, or skill challenges. Physical education may benefit from integration cognitive and affective dimensions alongside motor development, providing varied, engaging, and reflective movement experiences.^30^ This approach resonates with the ideas of cognitive activation^29^ and the capacity to act,^27^ emphasizing both operative and reflexive competencies that enable children to meaningfully interpret and engage in sport and physical activity.

### 4.1 Limitations

Several limitations should be acknowledged. First, the cross-sectional design precludes causal inferences and may bias the interpretation of indirect pathways.^63^^,^^64^ Although motor tests and questionnaire-based measures were assessed before parental reports of physical activity, the temporal separation between assessments resulted from organizational procedures within the same school year and does not allow conclusions regarding temporal or causal relationships. Longitudinal or intervention studies are needed to clarify temporal dynamics. Second, several measures relied on self-reports from children or parental reports of children's physical activity. While the applied measures have been validated for the relevant age groups, self-reported data may be affected by recall bias or limited introspective accuracy, particularly in younger children and regarding the cognitive dimension. Although parent-reported physical activity provides a complementary perspective, it may also be subject to reporting bias. Future research should consider combining subjective and objective assessments to provide a more comprehensive evaluation of physical activity behavior. Third, knowledge assessment requires further validation to confirm construct validity and reliability across contexts and age groups. While knowledge represents an important component of the cognitive dimension of physical literacy,^24^ the operationalization used in the present study does not fully capture broader cognitive processes involved in physical literacy, such as perceptual awareness or reasoning.^11^ Future studies should therefore examine whether additional indicators, including objective assessments of cognitive processes, provide further insights into the cognitive dimension. This aligns with broader calls to advance the assessment of the cognitive dimension within physical literacy, which remains a priority in frameworks such as the Global Physical Literacy Action Framework.^65^ Finally, the regionally specific sample may limit generalizability. Future studies should include more diverse samples and examine potential moderators such as socio-economic background, school type, or ethnic context.

## 5 Conclusions

Knowledge is a relevant yet small contributor to children's physical activity behavior. Declarative knowledge was moderately associated with overall active days, while procedural knowledge was linked to the number of sports and exercises performed primarily through physical and affective pathways. These findings highlight that knowledge exerts its relationship with physical activity behavior more strongly when embedded in embodied and motivational contexts, supported by the monistic nature of physical literacy. For practice, this implies that physical education should not restrict itself to movement execution but systematically integrate cognitive and affective elements, with particular attention to motivation and self-reflection. Future longitudinal studies are required to examine how these mechanisms develop over time.

## Ethics statement

All procedures performed in this study were in accordance with national law and the Declaration of Helsinki (1975, as revised). Ethics approval was obtained from the Research Ethics Committee at the University of Bayreuth (Approval No.: O 1305/1-GB).

## Informed consent to participate

Written informed consent was obtained from all participants and their legal guardians prior to participation. Participation was voluntary and could be withdrawn at any time. The participants’ parents received detailed written information about the study, along with its procedures and purpose.

## Data availability and reporting guidelines

The dataset supporting the conclusions of this article is available from the corresponding author on reasonable request. This study was reported according to the STROBE guidelines. The completed STROBE checklist is provided as supplementary material (Table S5).

## Author contributions

TS: Conceptualization, Data Curation, Formal Analysis, Funding acquisition, Investigation, Methodology, Writing – Original Draft, Writing – Review & Editing.

JC: Visualization, Interpretation & Discussion of Results, Writing – Review & Editing.

ST: Conceptualization, Methodology, Project Administration, Supervision, Writing – Review & Editing.

All authors contributed to the review and revision of the manuscript, and all authors have read and approved the final version of the manuscript.

## Funding statement

This study was part of the project *Bewegungswelt Oberfranken – active und bewegungskompetente Kinder* (*Movement World Upper Franconia – Active and physical literate children*) and was supported by the Oberfrankenstiftung (Funding code: FP01468).

## Declaration of competing interest

The authors declare that they have no known competing financial interests or personal relationships that could have appeared to influence the work reported in this paper.
