# Supplementary material for: Unveiling the role of knowledge within the concept of physical literacy and its empirical links to physical activity in children
Source: J Exerc Sci Fit. 2026 Sep 23;24(4):200524. doi: 10.1016/j.jesf.2026.200524 (PMC13651661; doi:10.1016/j.jesf.2026.200524)
Supplement: Supplementary file 1 [file mmc1.pdf]

Supplementary material

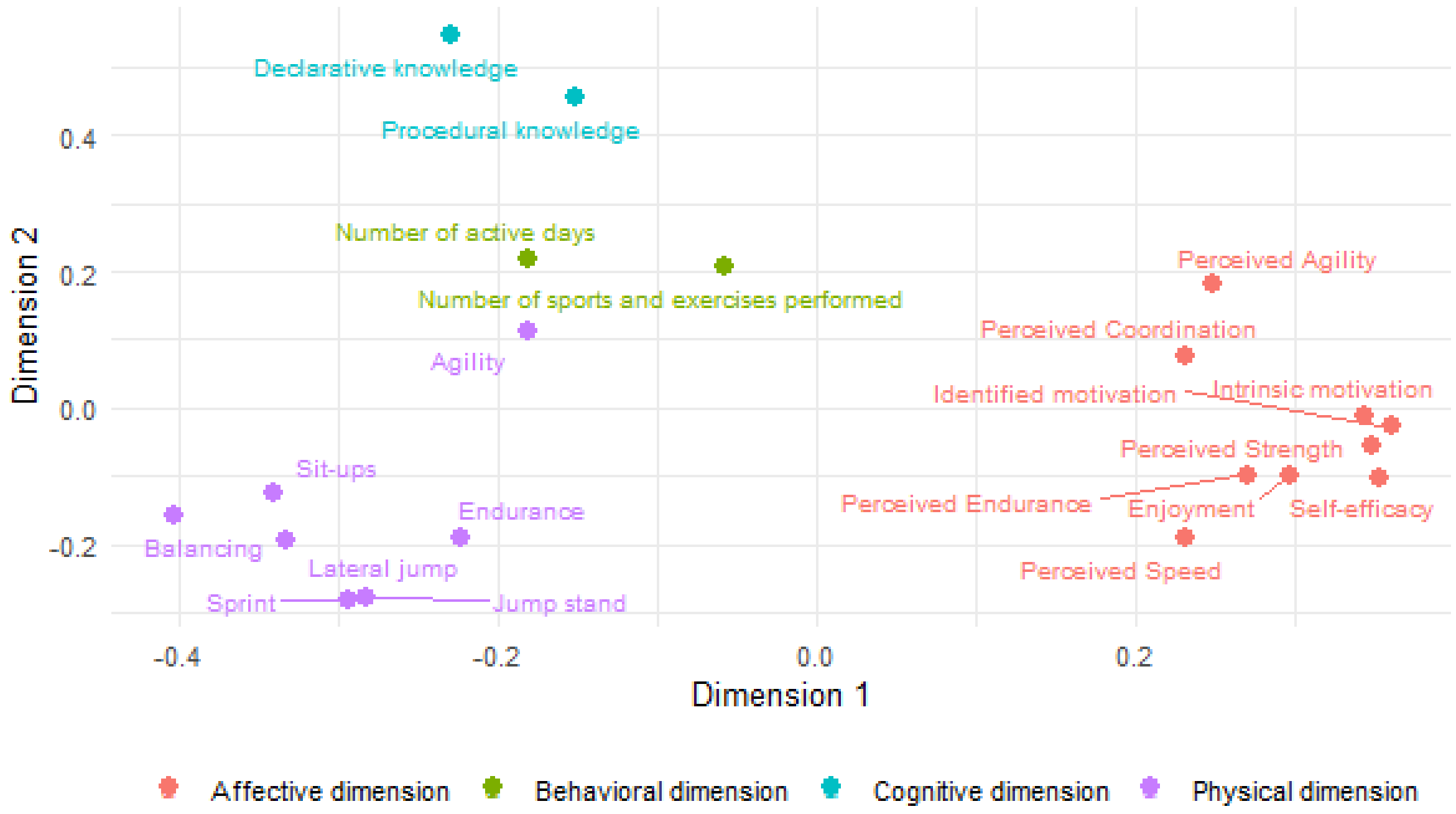

Supplementary Figure S1. MDS plot with all physical literacy dimensions.

Supplementary Table S1. Coding scheme of question about effect knowledge.

Title	Coding	Definition	Prototype example
At least two examples are mentioned	2	At least two examples are mentioned, referring to different response categories. The examples can also belong to the same response category.	"Because I'm out of breath. I'm sweating. I have sore muscles." (Physical reaction and physical adaptation)<br>"It's exhausting, but afterwards I feel good."<br>(Effort and well-being)
One example is mentioned	1	One example referring to a relevant response category is mentioned.	"I feel good." (Well-being)<br>"Because I'm running out of breath a little."<br>(Physical reaction)
Incorrect/ Unrelated/ Vague/ Missing	0	The question is answered with a response that does not match any relevant category, refers to an unrelated category (e.g., physical activity or affective components), or is too general, vague or missing.	Unrelated category:<br>"Riding a bike." (Physical activity)<br>"Because it's fun. (Affective component)<br>"Focused and happy. (Affective component)<br>"Based on my mood. (Affective component)<br><br>Too general, vague, incorrect or missing answers:<br>"I notice it always after sport." (Too general)<br>"I have a better feeling." (Too general)<br>"Feels good and I feel it." (Vague)<br>"Don't know." (No answer)

Supplementary Table S2. Categorization process of effect knowledge.

Relevant response categories	Protoype example
**Physical reaction**<br>= The question is answered with reference to physical reaction.	"I sweat." (Physical reaction)<br>"I'm hot. I'm out of breath." (Physical reaction)
**Physical adaptation**<br>= The question is answered with reference to physical adaptation.	"My stomach gets slimmer." (Physical adaptation)<br>"I get faster." (Physical adaptation)
**Effort**<br>= The question is answered in terms of the perceived effort.	"Because it's very exhausting." (Effort)<br>"Because I get tired afterwards." (Effort)
**Well-being**<br>= The question is answered in relation to well-being.	"I feel good." (Well-being)<br>"I feel relaxed." (Well-being)

Supplementary Table S3. Coding scheme of question about action knowledge.

Title	Coding	Definition	Prototype example
Training with a specification/ clarification	2	The response links training with time suggestion/commitment, social support, effort, or concentration; describes a movement and explicitly indicates improvement; or mentions a specific training principle.	"Ask Mom if she helps me. Train and try – never stop." (Social support and Training principle)<br>"I would keep my eyes only on the ball." (Training principle)<br>"I ask my friends, parents, and clubs." (Social support)<br>"Practice every day." (Time commitment)
General training	1	The answer refers only generally to training or practicing.	"Practice it." (General training)<br>" I would practice at home." (General training)
Incorrect/ Unrelated/ Vague/ Missing	0	The question is answered with a response that does not match any relevant category, refers to an unrelated category (e.g., desire for improvement or general physical activity), or is too general, vague or missing.	Unrelated category:<br>"I want to get better." (Desire for improvement)<br>"I want to improve in racing." (Desire for improvement)<br>"Climbing." (General physical activity)<br>"Move a lot." (General physical activity)<br>"Keep playing." (General physical activity)<br><br>Too general, vague, incorrect or missing answers:<br>"Learn many exercises." (Too vague)<br>"Shoot better." (Too vague)<br>"Don't know." (Missing answer)<br>"Learning." (Incorrect answer)

Supplementary Table S4. Categorization process of action knowledge.

Relevant response categories	Prototype example
**Social support**<br>= The question is answered with a reference to social support.	"I ask my friends, parents, and clubs." (Social support)<br>"Go to my parents." (Social support)
**Time commitment**<br>= The question is answered with a time-related statement.	"Practice every day." (Time commitment)<br>"Do more sports." (Time commitment)
**Effort**<br>= The question is answered with reference to effort or exertion.	"I would practice and try." (Effort)<br>"To give my best." (Effort)
**Concentration**<br>= The question refers to focus or attention.	"Do more sports and concentrate." (Concentration)<br>"Pay close attention. Don't mess around." (Concentration)
**Training principle**<br>= The question is answered using a specific training principle.	"I would keep my eyes only on the ball." (Training principle)<br>"I would first take a beginner course, then move up to higher groups." (Training principle)
**General training**<br>= The question is answered with general training statements.	"Practice it." (General training)<br>"I would practice at home." (General training)
**Movement description**<br>= The question is answered by describing a movement and its improvement.	"Run faster." (Movement description)<br>"Shoot a lot." (Movement description)

Supplementary Table S5. STROBE Statement – checklist of items that should be included in reports of observational studies.

	Item No.	Recommendation	Page No.	Relevant text from manuscript
**Title and abstract**	1	(*a*) Indicate the study's design with a commonly used term in the title or the abstract	1	The study design (cross-sectional study) is stated in the abstract.
		(*b*) Provide in the abstract an informative and balanced summary of what was done and what was found	1	The abstract summarizes the background, objectives, methods, main findings, and conclusions.
**Introduction**				
Background/rationale	2	Explain the scientific background and rationale for the investigation being reported	2 – 4	Scientific background and rationale, including physical literacy, cognitive domain, and knowledge concepts, are described.
Objectives	3	State specific objectives, including any prespecified hypotheses	4	Study objectives and hypotheses are explicitly stated.
**Methods**				
Study design	4	Present key elements of study design early in the paper	4 – 5	The cross-sectional study design is described in the Methods.
Setting	5	Describe the setting, locations, and relevant dates, including periods of recruitment, exposure, follow-up, and data collection	5	Study setting, participating schools, and period of data collection are described.
Participants	6	(*a*) *Cohort study*—Give the eligibility criteria, and the sources and methods of selection of participants. Describe methods of follow-up<br>*Case-control study*—Give the eligibility criteria, and the sources and methods of case ascertainment and control selection. Give the rationale for the choice of cases and controls<br>*Cross-sectional study*—Give the eligibility criteria, and the sources and methods of selection of participants	5	Eligibility criteria and participant recruitment are described.
		(*b*) *Cohort study*—For matched studies, give matching criteria and number of exposed and unexposed<br>*Case-control study*—For matched studies, give matching criteria and the number of controls per case		NA (This is not a cohort or a case-control study.)
Variables	7	Clearly define all outcomes, exposures, predictors, potential confounders, and effect modifiers. Give diagnostic criteria, if applicable	6 – 7	Outcome variables are clearly defined.
Data sources/ measurement	8*	For each variable of interest, give sources of data and details of methods of assessment (measurement). Describe comparability of assessment methods if there is more than one group	Table 1	Measurement instruments, scoring procedures, and data sources are described with references.

Bias	9	Describe any efforts to address potential sources of bias	12 – 13; 7 – 8	Potential sources of bias, including the cross-sectional design and self-reported measures, are discussed in the Limitations. Missing data were handled using FIML estimation.
Study size	10	Explain how the study size was arrived at	5	Sample size was not calculated a priori.
Statistical methods	12	(*a*) Describe all statistical methods, including those used to control for confounding	7 – 8	Statistical analyses, including structural equation modelling and estimation procedures, are described.
		(*b*) Describe any methods used to examine subgroups and interactions		NA
		(*c*) Explain how missing data were addressed	7 – 8	Missing data were handled using Full Information Maximum Likelihood (FIML).
		(*d*) *Cohort study*—If applicable, explain how loss to follow-up was addressed<br>*Case-control study*—If applicable, explain how matching of cases and controls was addressed<br>*Cross-sectional study*—If applicable, describe analytical methods taking account of sampling strategy		NA
		(*e*) Describe any sensitivity analyses		Due to the sample size, no further sensitivity analyses.
**Results**				
Participants	13*	(a) Report numbers of individuals at each stage of study—eg numbers potentially eligible, examined for eligibility, confirmed eligible, included in the study, completing follow-up, and analysed	4; Table 2	The final analytical sample is reported in the Results.
		(b) Give reasons for non-participation at each stage	5	Children absent on the assessment day or without parental consent were not included.
		(c) Consider use of a flow diagram		A flow diagram was considered but not perceived as carrying added value given the cross-sectional design.
Descriptive data	14*	(a) Give characteristics of study participants (eg demographic, clinical, social) and information on exposures and potential confounders	4; Table 2	Participant characteristics on page 4 and descriptive statistics in table 2 are presented.
		(b) Indicate number of participants with missing data for each variable of interest	7 – 8	Missing data handled using FIML.
		(c) *Cohort study*—Summarise follow-up time (eg, average and total amount)		NA (This is not a cohort study.)
Outcome data	15*	*Cohort study*—Report numbers of outcome events or summary measures over time		NA (This is not a cohort study.)

		Case-control study—Report numbers in each exposure category, or summary measures of exposure		NA (This is not a case-control study.)
		Cross-sectional study—Report numbers of outcome events or summary measures	Table 2 and Table 3	Summary measures of the outcome variables are reported.
Main results	16	(*a*) Give unadjusted estimates and, if applicable, confounder-adjusted estimates and their precision (eg, 95% confidence interval). Make clear which confounders were adjusted for and why they were included	Table 3	Standardized coefficients and 95% confidence intervals are reported.
		(*b*) Report category boundaries when continuous variables were categorized		NA (No categories for continuous variables were used.)
		(*c*) If relevant, consider translating estimates of relative risk into absolute risk for a meaningful time period		NA
**Discussion**				
Key results	18	Summarise key results with reference to study objectives	9 – 10	Key findings are summarized in first paragraph of Discussion.
Limitations	19	Discuss limitations of the study, taking into account sources of potential bias or imprecision. Discuss both direction and magnitude of any potential bias	12 – 13	Limitations of cross-sectional design, self-reported data, and generalizability are discussed.
Interpretation	20	Give a cautious overall interpretation of results considering objectives, limitations, multiplicity of analyses, results from similar studies, and other relevant evidence	9 – 13	Results are interpreted considering previous literature, study limitations, and implications.
Generalisability	21	Discuss the generalisability (external validity) of the study results	12 – 13	Generalizability is discussed in the Limitations.
**Other information**				
Funding	22	Give the source of funding and the role of the funders for the present study and, if applicable, for the original study on which the present article is based	14	Funding sources and the role of the funders are reported in the Funding Statement.

*Give information separately for cases and controls in case-control studies and, if applicable, for exposed and unexposed groups in cohort and cross-sectional studies.

Note: An Explanation and Elaboration article discusses each checklist item and gives methodological background and published examples of transparent reporting. The STROBE checklist is best used in conjunction with this article (freely available on the Web sites of PLoS Medicine at http://www.plosmedicine.org/, Annals of Internal Medicine at http://www.annals.org/, and Epidemiology at http://www.epidem.com/). Information on the STROBE Initiative is available at www.strobe-statement.org.
